# Enhancing Diagnostic Accuracy of Neurological Disorders Through Feature-Driven Multi-Class Classification with Machine Learning

**DOI:** 10.3390/diagnostics15172132

**Published:** 2025-08-23

**Authors:** Çiğdem Gülüzar Altıntop

**Affiliations:** Department of Biomedical Engineering, Faculty of Engineering, Erciyes University, Kayseri 38280, Türkiye; cigdemacer@erciyes.edu.tr

**Keywords:** neurological disorders, feature selection, electroencephalography, least absolute shrinkage and selection operator, machine learning, multi-class classification

## Abstract

**Background/Objectives:** Neurological disorders (ND) are a global health challenge, affecting millions and greatly reducing quality of life. Disorders such as Alzheimer’s disease, mild cognitive impairment (MCI), schizophrenia, and depression often share overlapping symptoms, complicating diagnosis and treatment. Early detection is crucial for timely intervention; however, traditional diagnostic methods rely on subjective assessments and costly imaging, which are not universally accessible. Addressing these challenges, this study investigates the classification of multiple ND using electroencephalography (EEG) signals. **Methods:** Various feature extraction methods were employed, and the Least Absolute Shrinkage and Selection Operator (Lasso) algorithm was utilized for effective feature selection. Two-class (disease–disease and healthy control–disease), three-class (healthy control and two ND, as well as three ND), and four-class (healthy control and three ND) classifications were conducted using different machine learning algorithms with the selected features. An EEG dataset comprising 40 Alzheimer’s patients, 43 healthy controls, 42 schizophrenia patients, 28 MCI patients, and 28 depression patients served as the experimental benchmark. **Results:** The Linear Discriminant Analysis (LDA) classifier achieved the highest accuracy, distinguishing between healthy controls and Alzheimer’s with 100% accuracy and demonstrating strong performance in other comparisons. Multi-class classification reached 84.67% accuracy for distinguishing depression, MCI, and schizophrenia, while four-class classification achieved 57.89%, highlighting the complexity of differentiating among multiple ND. The frequent selection of frontal lobe channels across ND indicates their critical role in classification. **Conclusions:** This study contributes to the literature by emphasizing disease-to-disease classification over the traditional control-versus-patient framework, highlighting the potential for more effective diagnostic tools in clinical settings.

## 1. Introduction

Neurological disorders (ND) encompass both central and peripheral nervous system diseases, including neurodevelopmental, neurodegenerative, and psychiatric conditions [1]. NDs are a primary cause of mortality and impaired quality of life worldwide. Early diagnosis can help reduce the course of many illnesses, if not totally eliminate them [2]. Common ND include Parkinson’s disease (PD), epilepsy, mild cognitive impairment (MCI), schizophrenia, and Alzheimer’s disease (AD), as well as cerebrovascular diseases like stroke, brain tumors, and developmental disorders like autism and attention deficit hyperactivity disorder (ADHD) [1]. In addition, mood disorders such as depression are regarded as major ND due to their substantial influence on cognitive and emotional processes. Depression, which is frequently associated with disorders such as Alzheimer’s and schizophrenia, is increasingly being explored for its neurological roots and consequences on brain function and structure [3].

These disorders can cause major complications, including memory loss and neurological malfunction, and have a significant impact on patients’ and families’ daily lives. As a result, early diagnosis is essential for timely treatment. However, early detection of diseases might be challenging. Current diagnostic methods, including clinical examination, neuropsychological assessment, and neuroimaging (MRI, fMRI, PET), are effective but often costly, time-consuming, and dependent on specialized expertise [4,5]. In regions with limited access to specialists, delays in diagnosis are common. Additionally, some imaging procedures involve invasive or radioactive materials, posing further risks. As a result, electroencephalography (EEG) has emerged as an attractive alternative due to its non-invasive nature, high temporal resolution, portability, and low cost [5,6]. EEG records brain electrical activity and can capture functional abnormalities across neurological and psychiatric disorders [7]. However, manual EEG interpretation is labor-intensive, prone to subjectivity, and challenged by the signal’s low signal-to-noise ratio and complexity [8].

To overcome these challenges, computer-aided diagnosis (CAD) systems using machine learning (ML) have been developed to automate EEG analysis. ML algorithms can identify patterns that may not be apparent to human observers, improving diagnostic accuracy and reducing interpretation time. Few research have explored developing a unified framework for multi-class classification of different ND. Most recent EEG-based studies have focused on binary classification of one ND (epilepsy [9], schizophrenia [10], AD [11], MCI [12], depression [13]) versus healthy controls. Deep learning methods, such as convolutional neural networks (CNNs) and recurrent neural networks (RNNs), have also been applied to EEG-based ND classification [14,15,16]. While these approaches can capture complex hierarchical patterns, they require large datasets for optimal performance, demand high computational resources, and often act as “black boxes” with limited interpretability [17]. These limitations make traditional ML approaches with engineered features more practical in many clinical settings, especially when datasets are small or medium-sized.

Table 1 summarizes representative EEG-based classification studies, highlighting the diversity in disorders, features, and classifiers, as well as the performance achieved.

Although many binary classification studies have achieved high performance, multi-class classification—which allows the simultaneous differentiation of multiple disorders—remains less explored. This limitation necessitates multiple CAD systems to cover different diseases, increasing cost and complexity. Most multi-class EEG studies are limited to three-class classification, such as AD vs. MCI vs. controls [24,25,26,27,28] or focus on specific neuropsychiatric disorders like depression and schizophrenia [29,30,31,32,33]. Disease-to-disease classification (e.g., AD vs. schizophrenia or AD vs. depression) is particularly rare. Nevertheless, to the best of my knowledge, there have not been any EEG-based studies that compare or classify AD and depression within a complete machine learning framework. Addressing these gaps might provide more understanding of the unique characteristics and similarities across these diseases, perhaps leading to more effective diagnostic and treatment methods. A unified framework for binary and multi-class classification of multiple NDs could improve diagnostic specificity, reduce cost, and enhance clinical applicability.

This work addresses this gap by proposing an ML framework for binary, three-class, and four-class classification of AD, depression, schizophrenia, MCI, and healthy controls. Features from time, frequency, entropy, and complexity measures were extracted, feature selection was applied to identify the most informative EEG channels, and performance was evaluated across multiple classifiers. Unlike studies in the literature, a multi-class classification of multiple disorders was proposed within the same framework. Beyond classification, it is investigated whether certain EEG channels could serve as potential biomarkers for these disorders. A cost-effective, interpretable, and scalable CAD system suitable for clinical environments with limited resources is aimed at being provided by this approach.

## 2. Methodology

**Figure 1:**
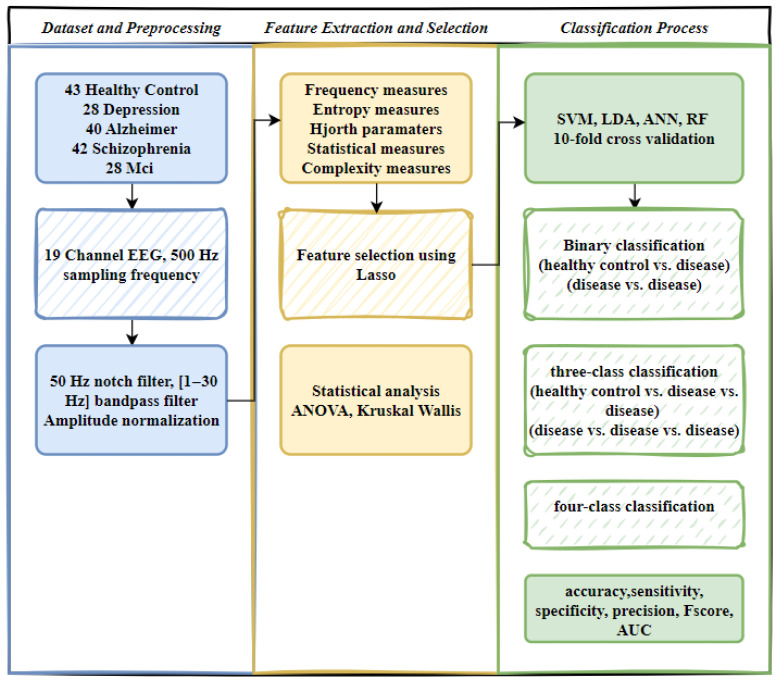
Workflow of EEG signal processing and classification.

In this study, EEG signals from four different ND and healthy controls were preprocessed, followed by feature extraction. After feature selection, two-class (control vs. disease, disease vs. disease), three-class (control vs. two diseases, three diseases), and four-class (control vs. three diseases, four diseases) classifications were conducted using various machine learning algorithms. Figure 1 illustrates the overall research framework.

### 2.1. Dataset

In this study, a publicly available dataset [34] containing EEG signals from AD, schizophrenia, MCI, depression patients, and control groups was used. EEG recordings were conducted in a standard clinical environment by a trained technician. Each participant underwent EEG monitoring between 8 AM and 1 PM, using a Nihon Kohden device with 19 electrodes arranged according to the international 10–20 system, with a sampling rate of 500 Hz. During the procedure, participants alternated between periods of rest with their eyes open and closed. Individuals who had undergone sleep EEGs were excluded from the study group. In the dataset, there were 230 subjects, including 28 with major depression, 42 with schizophrenia, 68 with cognitive impairment (40 AD, 28 MCI), and 95 controls. To ensure a balanced classification of the dataset, EEG signals of 43 randomly selected healthy controls were used. Table 2 presents demographic information for each neurological disease and for healthy controls.

This study was conducted without applying data augmentation techniques or generating synthetic samples. All analyses were performed exclusively on authentic EEG recordings to ensure that the results reflect genuine neurophysiological patterns.

### 2.2. EEG Signals and Preprocessing

**Figure 2:**
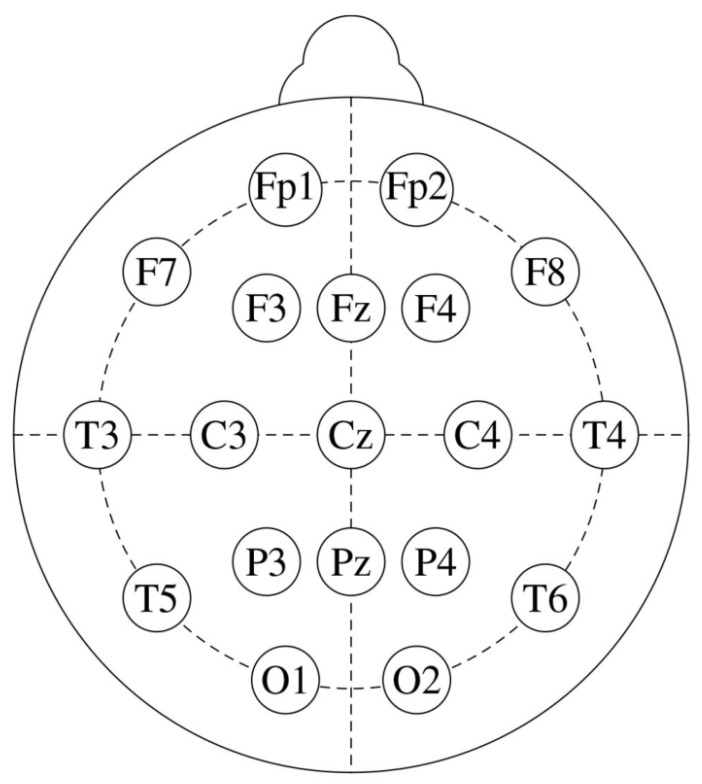
The standard 10–20 EEG electrode placements with 19 channels.

**Figure 3:**
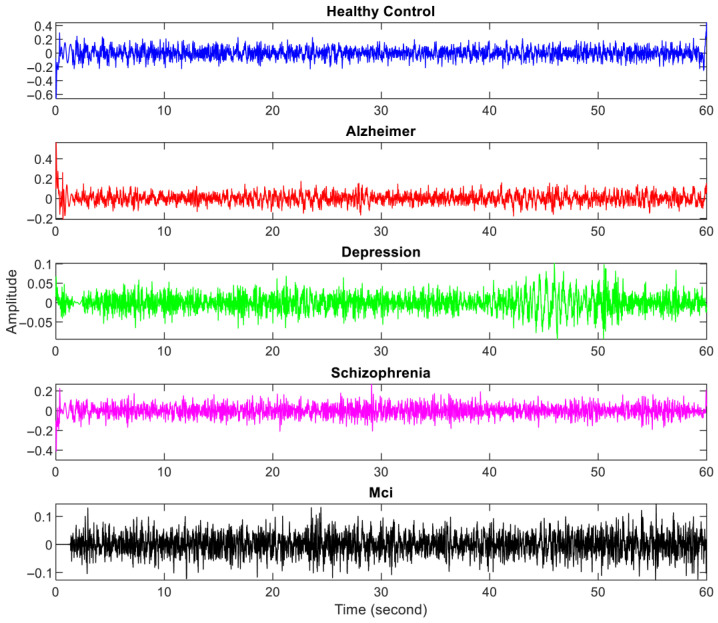
Filtered one-minute EEG signals from one person with each neurological disorder and healthy controls.

The amplitude of EEG recordings might vary depending on factors such as electrode location, skin impedance, and equipment variability. Normalization ensures that these differences do not affect the analysis, allowing for more consistent comparisons between subjects. Hence, the EEG signals were first normalized to a range between 0 and 1. To eliminate mains interference, a 50 Hz notch filter was applied. Afterward, a Butterworth bandpass filter [1–30 Hz bandpass] was used to filter the signals. The 10–20 international system is illustrated in Figure 2, while the specific electrode placements for each channel are detailed in Table 3. In Figure 3, the filtered EEG signals of the F7 channel are shown for a period of 1 min.

### 2.3. Feature Extraction

In the study, various features were extracted from EEG signals in the time axis and frequency axis to examine their distinctiveness in ND. While the features extracted in the time axis consist of statistical calculations, entropy values of different algorithms, and complexity measurements, calculations in the frequency axis are based on the results obtained from the power spectral density (PSD) analysis of frequency values and power values. PSD is a method for examining the frequency characteristics of a signal by evaluating how its power is distributed across different frequency bands [35]. This approach is particularly useful in EEG analysis, where it helps to identify dominant brainwave frequencies that are associated with various neurological states. By using the Fourier transform, PSD allows for a detailed analysis of the signal’s frequency content. Choosing an appropriate window length is essential to balance the trade-off between frequency resolution and variance in the estimation process [35]. The window length was heuristically selected at 512 after testing different values to achieve an optimal balance between frequency resolution and spectral estimation stability. Table 4 is provided to group the extracted features and briefly explain them with their abbreviations.

#### 2.3.1. Entropy Measures

In this study, instead of calculating a single entropy value, entropy values with different methodologies were calculated. Since entropy calculations are sensitive to length, calculating entropy by dividing the signal into certain windows and averaging the entropy matrix is a more accurate approach. Short windows may not accurately reflect slower processes, but lengthy windows may ignore faster alterations, affecting entropy estimates. For example, in EEG analysis, selecting a window that is too small might underestimate signal complexity, whereas an overly long window may smooth out crucial characteristics. Therefore, selecting a suitable window size is crucial for reliable entropy measurement [36,37]. In this study, after examining many studies, entropy calculation was performed for every two seconds of data (1000 samples) [38,39,40]. The entropy measurements are presented below with their formulations.

✓ Approximation Entropy (ApEn) [41] is a measure of signal complexity, particularly resistant to low-frequency noise. Higher ApEn values suggest greater complexity. Given a time series *X_N_* with *N* samples, subsets *p_m_*(*i*) are formed using *m* samples starting at the *i*-th position. Two subsets *p_m_*(*i*) and *p_m_*(*j*), are considered similar if the Euclidean distance between them is less than a threshold *r.* The set *p_m_* represents all patterns of length m within *X_N_*. The similarity calculation is formalized in Equations (1) and (2).

(1) $$\left|X_{i+k}-X_{j+k}\right|<r,\;0\leq k\leq m$$

(2) $$ApEn\left(m,r,N\right)=In\frac{C_{m}(r)}{C_{m+1}(r)}$$

(3) $$C_{i,m}\left(r\right)=\frac{n_{i,m}(r)}{N-m+1}$$

*n_i*,*m_*(*r*) represents the number of patterns in the set *p_m_* that are similar to *p_m_*(*i*), according to the similarity coefficient *r*. This calculation is performed for each pattern in *p_m_*. *C_m_*(*r*) is the average of all *C_i*,*m_*(*r*) values (see Equation (3)). For ApEn calculations, the parameters are usually chosen as *m* = 2 or *m* = 3, and *r* = 0.15 × *σ_X_* (*σ_X_* is the standard deviation of the original time series), or *r* = 0.25 × *σ_X_* or *r* = 0.2 × *σ_X_*. In this study, the embedding dimension is set to *m* = 2 and the similarity coefficient to *r* = 0.25 × *σ_X_*.

✓ Logenergy entropy (logenergy) [42] is predicted based on the energy present in the signal, which can result in higher entropy values compared to other algorithms. For a data series *X* with *N* samples, the energy of the signal and the logenergy value are calculated as follows:

(4) $$logenergy{(x}_{i})=\log\left(x_{i}^{2}\right)\;logenergy(x)=\sum_{i=1}^{n}\log\left(x_{i}^{2}\right)$$

✓ Unlike other entropy methods, Permutation Entropy (PermEn) [43] relies solely on the order of the time series amplitudes. This makes PermEn more computationally efficient [44]. The PermEn calculation is described by Equation (5).

(5) $$PermEn=-\sum_{i=1}^{N-(m-1)\tau }p_{i}\left(\pi \right)\log_{2}p_{i}\left(\pi \right)$$

In Equation (5), *p*(*π*) represents the relative frequency of each permutation *π*, with the embedding dimension *m* and lag *τ*. For the PermEn calculation, the embedding dimension was empirically set to six, and the lag was set to one. The PermEn value is 1 when all permutations are equally probable and decreases if the time series exhibits regularity.

✓ Renyi entropy (renyi) is an extension of Shannon Entropy [45]. The definition of renyi with a specific order is given in Equation (6). In this context, *p_i_* denotes the probability of the time series, and α ≠ 1 indicates the order. When α = 1, Renyi entropy simplifies to Shannon entropy. Renyi entropy remains consistent across various density functions.

(6) $${renyi}_{\propto }\left(x\right)=-\frac{\propto }{1-\propto }\sum \log_{2}p_{i}^{\propto }$$

✓ Tsallis entropy [46] for an EEG signal is derived from a generalized entropy measure. In Equation (7), *k* represents the length of the sub-dataset, *p_i_* is the probability function, and *q* is the Tsallis parameter (with *k* = 1000 and *q* = 2).

(7) $${tsallis}_{q}\left(x\right)=\frac{\sum_{i=1}^{k}(P_{i}-P_{i}^{q})}{q-1}$$

✓ Spectral entropy (spentropy) quantifies the unpredictability or complexity of power distribution across distinct frequency bands in a signal’s spectrum, providing information on the signal’s spectral properties. Spectral Entropy involves substituting the probability density function in Equation (5) with the power spectral density of the signal. It is calculated as follows

(8) $$spentropy\left(F_{i}\right)=-\sum_{i=fl}^{fh}F_{i\;}\log(F_{i\;})$$

where *F_i_* represents the normalized power in the *i*-th frequency band, and *f_l_* and *f_h_* represent the frequency bands.

#### 2.3.2. Hjorth Parameters

Hjorth parameters, introduced by Hjorth [47], include activity, mobility, and complexity. The activity parameter reflects the signal’s overall power: higher activity values indicate greater amounts of high-frequency components. Mobility is related to the standard deviation of the power spectrum, measuring how power distribution varies. Complexity assesses how much the signal deviates from a pure sine wave, with a maximum value of 1 indicating perfect similarity. The formulas for calculating Hjorth parameters are detailed in [47]. In this study, Hjorth parameters were calculated separately for the entire signal and for one-minute windows.

#### 2.3.3. Complexity Measures

Kolmogorov complexity [48], often known as algorithmic complexity, is the amount of information needed to describe a string or dataset in the most compact manner. It is defined as the length of the shortest program (in a certain programming language) that produces the supplied text when executed. Essentially, it measures the complexity of a string based on the shortest feasible explanation, indicating how intricate or simple the data is.

Lempel–Ziv complexity [49] measures the complexity of a sequence by measuring the number of unique substrings or patterns that may be seen while the sequence is processed gradually. It measures the sequence’s richness or regularity, indicating how much new information or variety is introduced as the sequence progresses. To calculate the Lempel value, the signal is first converted to a binary data array as in Equation (9). The threshold value *T* is determined by calculating the median or mean value of the time series (*x*(*n*)). In this study, the median value is used. Because the median is commonly selected as the threshold (*T*) due to its resistance to the effects of outliers. Its ability to remain stable despite extreme values makes it a reliable choice for setting thresholds [50].

(9) $$s\left(n\right)=\left\{\begin{array}{c} 1,\;\;if\;x\left(n\right)>T,\\ 0,\;\;if\;x\left(n\right)\leq T, \end{array}\right.$$

All features, except for frequency axis measures (freqmax, powmax), were calculated for signals divided into 2-s windows. The mean and variance of the resulting matrices were evaluated for each calculation (yielding the final feature value). Additionally, Hjorth parameters, kurtosis, and skewness values were obtained for 60-s windows across all signals. As a result, a total of 646 features were obtained (19 EEG channels × 34 features).

### 2.4. Feature Selection

In recent years, the volume of high-dimensional data accessible online has surged significantly. As a result, machine learning techniques face challenges in managing the vast array of input features, presenting an intriguing problem for researchers. To apply machine learning algorithms efficiently, data preprocessing is crucial. Among the various preprocessing techniques, feature selection stands out as one of the most vital and widely used methods, playing a key role in the success of machine learning algorithms [51]. Feature selection methods are grouped into three groups based on evaluation criteria and relation to the learning algorithm as the filtering technique, the Wrapper approach, and the embedded method [51]. Although each category has advantages and disadvantages, feature selection methods from each category have been used in biomedical image analysis and biomedical signal processing [52]. In recent research, mutual information-based feature selection [53], Recursive Feature Elimination (RFE) method [54], correlation-based feature selection (CBFS) [55], Kruskal–Wallis (KW) [56], and ReliefF [57] method are some of the methods used in the selection of features extracted from ND EEG signals. Since researchers continue to explore which feature selection method can be used and apply feature selection techniques tailored to the unique characteristics of each dataset, it suggests that the choice of method is largely based on experimentation. In this research, the Least Absolute Shrinkage and Selection Operator (Lasso) algorithm was chosen for feature selection through empirical testing. Lasso regularization [56] is an embedding approach based on the ℓ1-norm of a linear classifier’s coefficient in Equation (10).

(10) $$\hat{\beta }=\underset{\beta }{\underbrace{min}}c(\beta ,X)+\lambda \;\sum_{i=1}^{M}\left|\beta_{i}\right|$$

where $\sum_{i=1}^{M}\left|\beta_{i}\right|$ is regularization term, *M* is the number of features, λ shrinkage (regularization) parameter, and *c*(.) is the classification objective function.

### 2.5. Statistical Analysis

Selecting an appropriate statistical approach is crucial for analyzing biological data. Selecting the improper statistical approach might lead to interpretation issues and negatively impact study conclusions. For features that do not follow a normal distribution, non-parametric tests should be used, while parametric statistical tests are appropriate for features that follow a normal distribution [58]. Student’s *t*-test is applied to compare the means of two groups, whereas the ANOVA test, an extension of the *t*-test, is employed to compare the means across three or more groups. Non-parametric alternatives to these parametric methods exist as well. For instance, the Mann–Whitney U test serves as a substitute for the *t*-test, and the Kruskal–Wallis H test is the non-parametric counterpart to ANOVA [58]. Dunn’s test with Bonferroni correction was applied for non-parametric pairwise comparisons between independent groups to identify significant differences among ND. The Bonferroni adjustment is frequently used to control the family-wise error rate. For parametric pairwise comparisons, Tukey’s test was utilized to detect significant differences in ND. Tukey’s test is widely used in biomedical research and is especially appropriate when group sizes are balanced [59]. In this study, the R statistics software (R 4.4.2 version) was used for statistical analysis processes.

### 2.6. Machine Learning Algorithms

In this research, features derived from EEG signals were classified using several ML models, including SVM, LDA, ANN, and RF algorithms. The classification process was conducted separately for two-class (healthy control vs. disease, disease vs. disease), three-class, and four-class classification to assess their ability to distinguish ND. The classifiers were trained using features selected through the Lasso method. The model performance was evaluated using 10-fold cross-validation. In the 10-fold cross-validation, 90% of the data was used for training, while the remaining 10% served as the test set, with the procedure repeated for each fold. Care was taken to maintain class balance during k-fold cross-validation. Preserving class proportions in each fold helps reduce the risk of overfitting to certain classes, especially in small datasets. Performance metrics were calculated at each iteration.

SVM is a widely used non-probabilistic classifier that operates by finding a decision boundary, or hyperplane, that maximizes the margin between support vectors belonging to different classes. For linearly separable data, SVM uses hyperplanes in an N-dimensional space to distinguish between different feature groups. Kernel functions are employed when the data is not linearly separable, mapping it to a higher-dimensional space to facilitate classification [60].

RF is an ensemble learning technique that uses numerous decision trees to increase accuracy while minimizing overfitting. It works by training several trees on random samples of data and averaging their predictions, making it reliable and effective for classification and regression applications [61]. LDA is a statistical technique for dimensionality reduction and classification. It selects a linear combination of characteristics that best distinguishes several classes by maximizing the distance between the means of distinct classes while minimizing variation within each [62].

ANN consists of three key layers: the input layer, one or more hidden layers, and the output layer. The number of neurons in the input layer is equal to the number of features representing the classified objects, while the output layer’s neurons correspond to the total number of classes [63]. In a fully connected backpropagation network, each neuron in the hidden and output layers is connected to every neuron in the preceding layer through a set of numerical weights. The training process allows the network to learn the relationship between inputs and outputs, using backpropagation as a common supervised learning technique. Training data includes pairs of inputs and their associated outputs, and the network adjusts its weights and biases to minimize prediction errors on the training set [63].

In this study, the MATLAB (2024a version) program was used for feature extraction, feature selection, and the classification process.

### 2.7. Evaluation Metrics

This study’s evaluation metrics include mostly used metrics: accuracy, sensitivity, specificity, precision, F-score, and Area Under the Curve (AUC) for the Receiver Operating Characteristic (ROC) curve [64].

## 3. Experimental Results

In this section, the results of feature selection, statistical analysis, and classification studies are given.

### 3.1. Feature Selection Results

**Figure 4:**
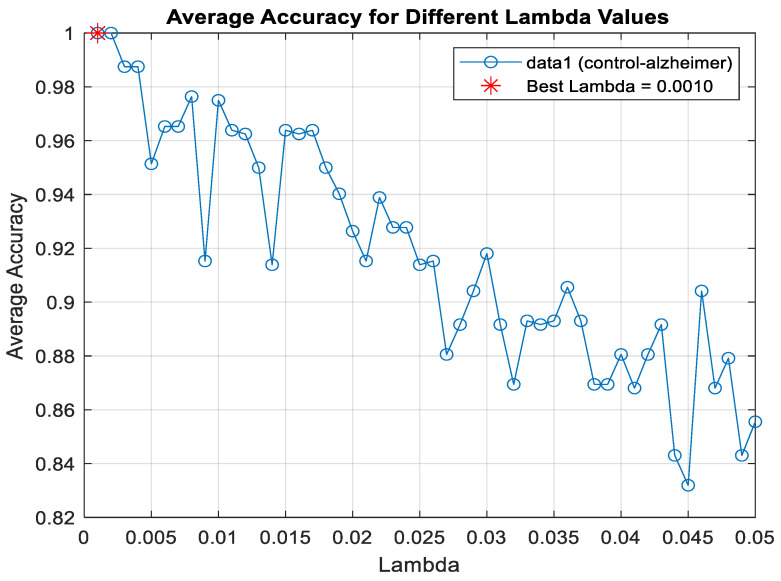
Lambda value determination for classification healthy control and AD using LDA algorithm.

**Figure 5:**
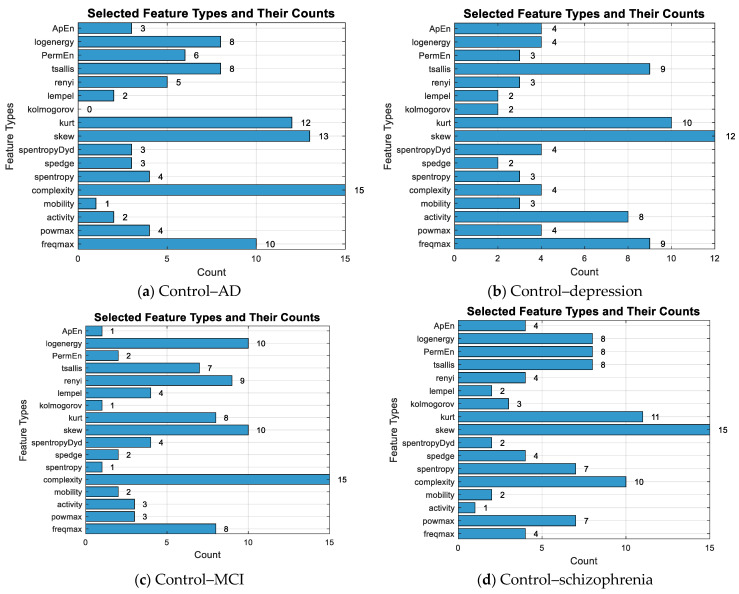
The number of selected features for the binary classification and the total count of selected features.

The Lasso algorithm was used for feature selection to reduce the data burden and improve classification performance. However, determining the optimal value of the lambda (λ) parameter in Lasso poses a challenge. Therefore, the lambda value was iteratively changed starting from 0.001 up to 0.05, and the lambda value that yielded the highest classification accuracy was selected, as seen in Figure 4. So, the shrinkage parameter (λ) was examined with 10-fold cross-validation, and the value with the lowest cross-validation loss (λ = 0.001) was chosen. Information about the features selected for binary classification was given in Figure 5. The number of features selected for two-class classification (binary classification) was given in Table 5.

**Figure 6:**
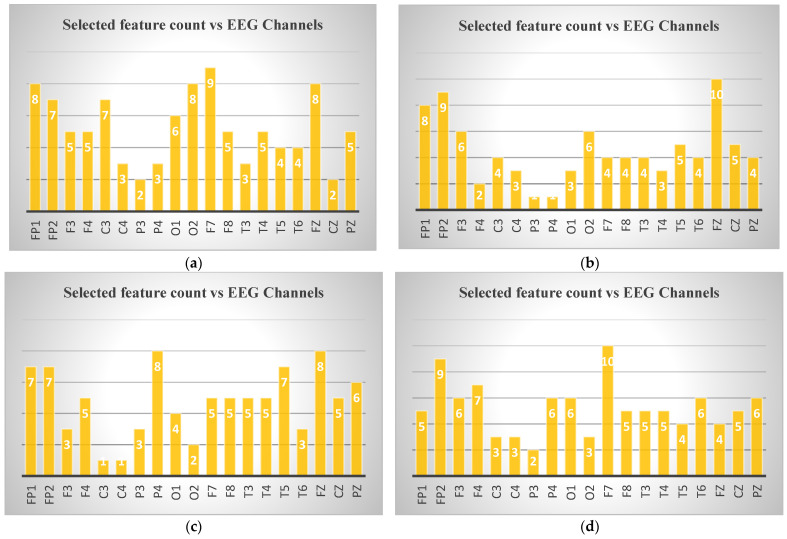
Selected feature counts for binary classification according to EEG channels: (**a**) control–Alzheimer, (**b**) control–depression (**c**) control–MCI, (**d**) control–schizophrenia.

In Figure 6, the numbers of features selected for binary classification according to channels were given. In the examination of EEG channel selections for different ND, specific channels emerged for each disease. For AD, channel F7 had the highest number of selected features (9), followed by O2 (8 features), indicating its importance in distinguishing this disorder. In depression, Fp2 and Fz showed significant relevance, with Fp2 having 9 features and Fz having 10 features. For MCI, P4 was the most significant channel with 8 selected features, followed by Fp1 and Fz, both showing 7 features. Finally, in schizophrenia, F7 again had the highest feature count with 10 selected features, followed by Fp2 and F4 (9 and 7 features). These channels highlight critical brain regions for classifying these disorders.

### 3.2. Statistical Analysis Results

**Figure 7:**
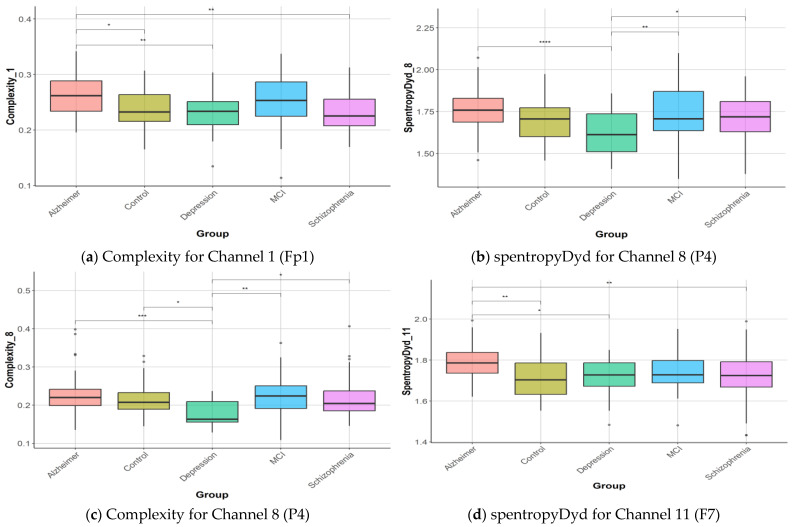
Box-plot graphics for ND and healthy control. Statistical significance between groups is indicated by asterisks (* *p* < 0.05; ** *p* < 0.01; *** *p* < 0.001; **** *p* < 0.0001).

The discrimination of features among the five groups (healthy controls, AD, depression, MCI, and schizophrenia) was assessed using both ANOVA and Kruskal–Wallis tests. Following these tests, post hoc analyses were conducted to determine which groups differed significantly from each other. The detailed results, including specific group comparisons and statistical significance, are provided in Supplementary Files for further reference. As seen in Supplementary File S1, 239 of the features yielded results that created a significant difference between the groups (*p* < 0.05). The post hoc analysis revealed that AD exhibited significant differences 184 times compared to other groups. The control group showed differences 151 times, depression in 119, schizophrenia in 52, and MCI in 28. These findings highlight the varying degrees of distinction between the conditions across different features, which are detailed further in the Supplementary File S1. Box-plot graphs for complexity and spentropyDyd, which are some of the features that reveal the most differences between the groups, were given in Figure 7. As seen in Figure 7, the complexity feature of the 8th EEG channel created a significant difference between the AD–depression, depression–control, depression–MCI, and depression–schizophrenia groups. The post hoc analysis results for the 4 features in Figure 7 according to the Tukey test were given in Supplementary File S2.

### 3.3. Parameter Tuning and Model Performance Timings

In the parameter tuning process for machine learning algorithms, a Bayesian optimization approach has been employed to efficiently explore the hyperparameter space. To ensure robust evaluation, a 10-fold cross-validation method was used to partition the data into training and test sets. Training and testing times of the algorithms were recorded. This approach not only provided a comprehensive assessment of model performance but also enabled a detailed comparison of algorithms in terms of their training and testing durations. By analyzing these timings, insights were gained into the computational efficiency of each algorithm, highlighting the trade-offs between parameter optimization and execution speed. As shown in Table 6, the running times of the algorithms for two-class classification were evaluated to compare their computational efficiency.

Upon analyzing the training times, it is evident that RF has the shortest training time across all diseases, taking less than a second, which is significantly quicker than the other algorithms like SVM, LDA, and ANN. On the other hand, LDA also shows relatively fast training times, though slightly longer than RF. ANN, while exhibiting the slowest training times, particularly for AD, outperforms others in testing time, showing consistently faster results in comparison to SVM, LDA, and RF, especially for quick evaluations. These results suggest that LDA is efficient for both training and testing, while ANN takes longer to train but is very fast in testing.

### 3.4. Classification Results

In this study, the performance of various classification algorithms (SVM, LDA, ANN, RF) for the classification of ND using features extracted from EEG signals was evaluated with 10-fold cross-validation. Classification results are presented in tables as mean ± standard deviation of each fold of 10-fold cross-validation. In Table 7, the performance measures of two-class classification (binary classification) according to control–disease classification were given. Table 8 shows the results of binary classification for disease–disease classification. The classification results for control–disease and disease–disease comparisons reveal varying levels of effectiveness across machine learning models like SVM, LDA, ANN, and RF. In the *control–AD* classification, LDA performed exceptionally well, achieving perfect scores for accuracy (1.0000 ± 0.0000), sensitivity, specificity, precision, F1 score, and AUC. This indicates that the model is perfectly classified between the control and AD groups across all metrics. In contrast, RF performed significantly worse, with lower accuracy (0.7208 ± 0.1498), and only moderate AUC (0.7225 ± 0.1474). This pattern highlights LDA’s robustness in distinguishing AD compared to other models. For the *control–depression* comparison, SVM delivered a high accuracy of 0.9589 ± 0.0945 and an impressive AUC of 0.9917 ± 0.0264, making it highly reliable in this classification. LDA also showed excellent results, with an accuracy of 0.9857 ± 0.0452 and similar performance in sensitivity and specificity. RF again underperformed with an accuracy of 0.7339 ± 0.1186, showing reduced sensitivity but moderate specificity (0.8550 ± 0.2088). The results emphasize how LDA and SVM succeed in capturing both the true positives (sensitivity) and true negatives (specificity) in the depression classification. In the *control–schizophrenia* comparison, both LDA and SVM provided consistently strong performances. LDA achieved an accuracy of 0.9778 ± 0.0468, and SVM had 0.9403 ± 0.1010, with high AUC values for both models (0.9800 ± 0.0422 and 0.9738 ± 0.0641, respectively). Meanwhile, RF continued to lag behind, offering an accuracy of 0.7278 ± 0.1386, reflecting its challenges in accurately distinguishing schizophrenia from the control group. In the *control*–MCI classification, LDA maintained strong performance with an accuracy of 0.9714 ± 0.0602, indicating its high reliability in distinguishing control subjects from MCI patients. SVM and ANN followed closely with the same accuracy of 0.9571, and SVM had a high AUC value (1.0000 ± 0.0000). In comparison, RF lagged behind, showing lower accuracy (0.7304 ± 0.1589) and reduced sensitivity, again confirming its relative inefficiency in handling neurological disorder classifications. Overall, LDA excelled in this control–MCI distinction. These findings underline LDA’s superior generalizability across various ND.

In the disease–disease classification, particularly for the *AD–MCI* comparison, LDA remained highly effective with 0.9690 ± 0.0655 accuracy, further supported by perfect specificity and precision (1.0000 ± 0.0000). Similarly, SVM and ANN yielded high accuracies (0.9262 ± 0.0781) but still underperformed compared to LDA. RF, once again, demonstrated lower efficiency, showing variability across metrics, particularly in specificity and AUC. For the *AD–schizophrenia* classification, LDA performed well with 0.9278 ± 0.0624 accuracy and balanced sensitivity/specificity. Although SVM showed an accuracy of 0.8667 ± 0.1220, the model’s AUC was still relatively strong (0.9238 ± 0.1256). ANN outperformed SVM with an accuracy of 0.9028 ± 0.1143. RF, however, had the lowest accuracy (0.7181 ± 0.1058), confirming the trend of reduced effectiveness compared to other methods. For the *depression–AD* classification, LDA achieved an accuracy of 0.9429 ± 0.0738, demonstrating solid performance across most metrics. SVM followed with an accuracy of 0.8810 ± 0.0635, and ANN closely matched with 0.8238 ± 0.1118, showing comparable AUCs. RF, however, had lower accuracy at 0.7333 ± 0.1830, confirming its relative inefficiency. In the *depression–MCI* classification, SVM achieved the highest performance with an accuracy of 0.9667 ± 0.0703, displaying excellent consistency across other metrics like specificity and precision (0.9667 ± 0.1054 for both). LDA followed closely with an accuracy of 0.9633 ± 0.0777, showing perfect precision and specificity (1.0000 ± 0.0000). ANN performed comparably with an accuracy of 0.9467 ± 0.0864, while RF struggled with an accuracy of 0.6700 ± 0.2457, highlighting its lower effectiveness in this classification task. In the *schizophrenia–MCI* classification, LDA outperformed other models with an accuracy of 0.9571 ± 0.0690, along with strong specificity and precision (0.9750 ± 0.0791 for both). SVM showed a slightly lower accuracy of 0.8714 ± 0.1251 but still delivered reliable precision (0.9000 ± 0.1748). ANN underperformed compared to the others, with an accuracy of 0.7857 ± 0.2156, reflecting its variability. RF also showed moderate performance with an accuracy of 0.7429 ± 0.1622, lagging behind in both sensitivity and specificity compared to LDA and SVM. Similarly, in the *depression–schizophrenia* comparison, LDA excelled, showing an accuracy of 0.9571 ± 0.0690 and nearly perfect AUC, indicating a strong ability to differentiate between these disorders. RF’s performance remained significantly lower, with an accuracy of 0.6857 ± 0.1475 and sensitivity of 0.4333 ± 0.3063, confirming its struggle with disease-to-disease distinctions.

Overall, LDA consistently delivered the highest classification performance in both control–disease and disease–disease comparisons, across multiple ND. SVM also performed well, especially in distinguishing depression and AD from the control group. ANN, similarly to SVM, demonstrated effective performance in most cases, offering a viable alternative for classification. RF, on the other hand, displayed the weakest performance in most cases, with lower accuracy, sensitivity, and AUC scores, suggesting that it may not be as suitable for these medical classifications. These results indicate that LDA and SVM are generally better suited for binary classifications involving ND, while RF may require optimization to improve its performance in these contexts.

In the disease–disease classification, AD-MCI was the best-performing classification, with LDA achieving the highest accuracy of 0.9690 ± 0.0655. This result was reinforced by perfect specificity and precision, making LDA the most reliable model in this context. Similarly, for the Depression-MCI comparison, SVM stood out with an accuracy of 0.9667 ± 0.0703, demonstrating its consistency across various metrics like sensitivity, specificity, and AUC, indicating its strong capability in differentiating between these two conditions effectively.

Table 9 and Table 10 present the results of the three-class classification using the LDA algorithm. Since the LDA algorithm is more successful than the others, only the results of the classification with LDA were given for the 3-class and 4-class classification. In the three-class classification results for the LDA algorithm, control–MCI–AD achieved the highest accuracy at 0.7932 ± 0.1041, along with strong sensitivity and specificity values. Among the disorder-only groups, the depression–MCI–schizophrenia classification performed best, with 0.8467 ± 0.1088 accuracy and a high AUC of 0.9128 ± 0.1090, indicating effective separation between these three disorders. In contrast, the depression–schizophrenia–AD group showed the lowest performance, with accuracy dropping to 0.6455 ± 0.1088 and lower precision and F-score values. In Table 9 and Table 10, the values highlighted in red correspond to the best-performing results among the evaluated algorithms.

Table 11 presents the results of the four-class classification using the LDA algorithm. The classification accuracy varied across different combinations of classes. The highest accuracy was observed in the control–depression–schizophrenia–cognitive decline classification, achieving an accuracy of 0.5789 (±0.1309). This was followed closely by the control–depression–schizophrenia–MCI classification, with an accuracy of 0.5605 (±0.1410). The control–depression–schizophrenia–AD classification exhibited a lower accuracy of 0.5146 (±0.1148), indicating challenges in distinguishing between these conditions. The lowest accuracy was recorded in the depression–schizophrenia–AD–MCI classification, at 0.4857 (±0.1171). Across all classifications for four-class, sensitivity, specificity, precision, and F-score values also reflected the varying degrees of model performance, highlighting the complexities in multi-class classification tasks.

**Figure 8:**
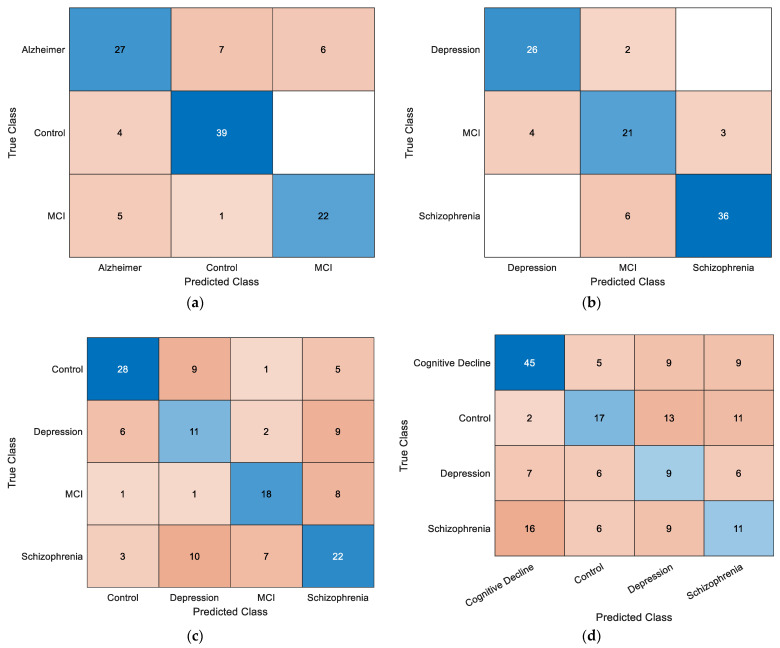
Overall confusion matrices obtained using the LDA classifier for (**a**) control–AD–MCI, (**b**) depression–MCI–schizophrenia, (**c**) control–depression–schizophrenia–MCI, and (**d**) control–depression–schizophrenia–cognitive decline classifications.

The confusion matrices in Figure 8 illustrate the classification performance of the LDA model across four experimental scenarios. In the control–AD–MCI task (a), the control class achieved the highest correct classification rate, whereas partial misclassification occurred between AD and MCI. In the depression–MCI–schizophrenia task (b), schizophrenia was identified with high accuracy, while MCI and depression exhibited greater overlap. The four-class control–depression–schizophrenia–MCI scenario (c) proved more challenging, with notable misclassification between depression and schizophrenia. Similarly, in the control–depression–schizophrenia–cognitive decline scenario (d), confusion was observed between Control and cognitive decline, as well as between depression and schizophrenia. Overall, multi-class configurations with more than three categories showed increased misclassification rates, reflecting the higher complexity and feature overlap among certain neurological disorders.

## 4. Discussion

Clinical EEG diagnosis presents several challenges: firstly, the accuracy of diagnoses is largely contingent upon the expertise of highly trained EEG specialists. Secondly, acquiring the skills necessary to interpret EEG recordings requires extensive pathological education over several years. Lastly, the process of analyzing EEG data is labor-intensive and can be both time-consuming and mentally taxing [65]. Therefore, there is a growing need for a computer-based system capable of automatically diagnosing multiple ND, which would streamline the process, reduce the burden on specialists, and improve diagnostic accuracy.

Very limited research on ND uses nonlinear EEG analytic techniques. In most studies, linear approaches such as power spectral density and frequency analysis have been used more often. Nonlinear approaches, on the other hand, are gaining popularity due to their ability to find more complicated dynamics in brain signals, providing crucial insights that linear methods may overlook, particularly when discriminating between various neurological diseases. The EEG signals may also be analyzed in a nonlinear way, which makes it possible to obtain an understanding that is not given by the linear measures. For example, Higuchi’s fractal dimension (HFD) has been shown to reflect higher complexity inside depressed patients as compared to healthy control individuals in all brain areas. Such studies have reported such significant success in classifying depression that an enhanced probabilistic neural network model has achieved 91.3% accuracy based on seven frontal EEG channels, indicating its possible applicability in the clinical field for mental health diagnostics [66]. Also, The Lempel–Ziv complexity (LZC) of multi-channel resting EEG has been shown to be effective in evaluating a variety of neurological and mental diseases, including serious depression [67,68].

Numerous studies show a significant correlation between cognitive decline and the reduction in EEG irregularity [40,69]. For this reason, in this study, both frequency axis and time axis (entropy, statistical measures) features are extracted from EEG signals. Although previous research suggests a framework for multi-class EEG classification, to the best of my knowledge, no studies have used the machine learning model to classify more than two diseases in healthy people and disease–disease classification. The aim of this study is to develop a system capable of automatically detecting four ND—Alzheimer, schizophrenia, depression, and MCI—using EEG signals, improving diagnostic speed and accuracy. Following this context, some studies in the literature are shown in Table 12.

Comparing the present results with selected studies in Table 12 the proposed LDA model achieved notably higher accuracy in several cases. For instance, in the classification of schizophrenia and depression, this study reached 95.71% accuracy with 95% sensitivity and specificity, outperforming the results of Jang et al. [71]. In the work of Wang et al. [33], a 10-fold cross-validation approach with LDA yielded 74.32% accuracy, which is lower than the performance observed in the present study for similar classification tasks. Likewise, when compared with the results of Hassanzadeh et al. [73], the proposed method achieved 92.78% accuracy in the same two-class classification and 76.03% accuracy in the equivalent three-class classification, both exceeding the accuracies reported in their work.

Also, in this study, the LDA classifier produced accuracies of 100% for control–AD, 97.14% for control–MCI, 96.90% for MCI-AD, and 79.32% for 3-class classification (AD–MCI–control), respectively. This performance is consistent with previous EEG-based ND classification studies, where accuracies typically ranged between 70% and 98% depending on the dataset size, feature set, and classification task. For example, Huang et al. [70] reported 88.2% accuracy in distinguishing depression from healthy controls using KNN after Lasso-based feature selection. Wang et al. [33] achieved 79.27% accuracy in a three-class classification of schizophrenia, depression, and healthy controls using a convolutional neural network (MUCHf-Net). Cheng et al. [72] applied dynamic functional connectivity features with a random forest model to classify four groups (nonpsychotic major depression, psychotic major depression, schizophrenia, and healthy controls), obtaining 73.1% accuracy. Compared to these works, the present study’s LDA approach demonstrated competitive or superior performance in both binary and multi-class settings while using a broader classification framework encompassing multiple ND combinations.

According to Table 7 and Table 8, SVM also performed strongly, particularly in control–depression classification, with an accuracy of 0.9589 ± 0.0945 and an AUC of 0.9917 ± 0.0264. In the control–schizophrenia classification, the ANN method outperforms SVM. Specifically, ANN achieves an accuracy of 0.9639 ± 0.0583, while SVM shows an accuracy of 0.9403 ± 0.1010. However, RF exhibited weaker results, with its accuracy ranging from 0.6700 to 0.7524, depending on the comparison, often underperforming in sensitivity and AUC scores.

In the disease–disease comparisons, similar trends were observed. LDA consistently achieved high accuracy, sensitivity, and specificity values, especially in the Alzheimer–MCI comparison (accuracy of 0.9690 ± 0.0655, AUC of 0.9750 ± 0.0527). SVM also showed solid performance, with accuracy values exceeding 0.90 in most comparisons. Although the ANN method generally performs well, it falls short of LDA in some classifications, particularly in the depression–MCI comparison, where ANN shows an impressive accuracy of 0.9467 ± 0.0864. However, RF again struggled, with accuracy values ranging from 0.6857 to 0.7524 and often demonstrating lower sensitivity and AUC values compared to LDA, ANN, and SVM.

The results from Table 9 and Table 10 demonstrate the performance of the LDA algorithm in three-class classification tasks involving various ND. In the control–disease–disease classification (Table 9), the highest accuracy was observed in the control–MCI–Alzheimer classification, with an accuracy of 0.7932 ± 0.1041, highlighting the model’s strength in distinguishing between these conditions. Conversely, the lowest performance was seen in the control–depression–MCI classification, with an accuracy of 0.7114 ± 0.1513. In the three disorders comparison (Table 10), the depression–MCI–schizophrenia classification yielded the highest accuracy of 0.8467 ± 0.1088, indicating effective differentiation among these disorders.

The results of the four-class classification indicate variability in the LDA algorithm’s performance across different combinations. The highest accuracy was achieved in the control–depression–schizophrenia–cognitive decline classification (57.89%), while the lowest accuracy was found in the depression–schizophrenia–Alzheimer–MCI classification (48.57%). These results highlight the challenges in distinguishing between certain conditions and demonstrate significant differences in model performance. Overall, the sensitivity, specificity, and accuracy values reflect the complexities involved in multi-class classification tasks.

Overall, LDA proved to be the most effective classification method across the board, while RF demonstrated significant variability and generally lower performance across different metrics. The RF classifier consistently showed lower performance across classification tasks. This may reflect the combination of a relatively small dataset, high feature dimensionality, and a reduced number of samples per class. Although hyperparameters such as the number of trees were optimized, these factors may have limited the classifier’s generalizability. Future studies should consider alternative balancing techniques, feature reduction strategies, or ensemble approaches to enhance RF performance.

According to the statistical analysis results, as seen in Figure 6, the channel Fp1 is selected for all four ND (Alzheimer’s, depression, MCI, and schizophrenia). While the number of features selected for this channel varies, its consistent appearance across all conditions suggests its potential importance in distinguishing these disorders. Additionally, other channels like Fp2, F7, and Fz are frequently selected, though not universally across all disorders. These shared channels might provide valuable insights for comparative analysis in multi-class classification tasks.

The frequent selection of frontal lobe channels (Fp1, Fp2, F3, F7, Fz) across the different ND in the Figure 6 suggests that frontal regions of the brain may play a critical role in differentiating these disorders. The frontal lobe is responsible for higher cognitive functions such as decision-making, attention, and emotional regulation, which are often impaired in these disorders. This focus on frontal channels could indicate that EEG signals from this region are particularly informative for identifying changes in brain activity associated with these conditions. Many neurological and psychiatric disorders studied (e.g., depression, Alzheimer’s, schizophrenia, MCI) involve functional alterations in frontal lobe networks, which are reflected in EEG measures of complexity, entropy, and spectral power [75,78,79,80]. Prior studies have also shown that frontal EEG abnormalities are robust biomarkers in these conditions, supporting this study’s findings [75,78,79,80]. However, further work with larger and more diverse datasets is needed to confirm the generalizability of these results.

## 5. Limitations and Future Work

This study is limited by a class imbalance problem. Class imbalance is a well-known challenge in EEG-based classification tasks, as it can bias model training and reduce generalizability. While oversampling methods such as SMOTE and ADASYN are commonly employed in general machine learning research, recent studies have highlighted potential pitfalls in biomedical contexts. Bunterngchit et al. [81] emphasize the importance of carefully imbalance-handling techniques to preserve the neurophysiological validity of EEG signals. Similarly, Rasool et al. [82] review deep learning-based strategies for addressing class imbalance, noting that although synthetic data generation can improve performance in some cases, it may also introduce artifacts that reduce clinical reliability. In this study, to maintain the authenticity of EEG recordings and avoid the potential biases of synthetic samples, only real, experimentally acquired data were used. Another limitation of this study is the absence of detailed clinical characterization of the participant groups. Key information, such as disease stage, medication status, comorbid conditions, and other relevant clinical variables, was not available, which may have influenced EEG patterns. Future studies should incorporate these factors to enhance the interpretability and generalizability of the findings. Also, in future studies, the analysis will be expanded to include other ND, such as epilepsy, to further investigate classification capabilities.

## 6. Conclusions

In this study, various features were extracted from EEG signals to achieve high-accuracy classification of multiple ND and to investigate the differentiation among these disorders. Feature selection was conducted using the Lasso algorithm, followed by classification using SVM, LDA, ANN, and RF algorithms. The results revealed that in binary classification, AD was accurately classified with 100% accuracy from healthy controls, while the Alzheimer–MCI classification achieved an accuracy of 96.90%. In the three-class classification involving depression, MCI, and schizophrenia, an accuracy of 84.67% was obtained. However, in the four-class classification, the maximum accuracy reached 57.89%. These findings highlight the effectiveness of EEG signal analysis in diagnosing and differentiating multiple ND. Furthermore, this research contributes to the field by utilizing a unique dataset that has not been previously examined in relation to these disorders in this manner. In conclusion, this study contributes to the literature by focusing on disease-to-disease classification and multi-class differentiation among ND, rather than control vs. patient classification.

## Figures and Tables

**Table 1:** Some binary classification studies.

Ref.	Disorder(s)	Feature Extraction and Selection	Classifier(s)	Accuracy
[18]	AD vs. Control	Frequency axis features, power, coherence, power ratio, frequency bandwidth; SVM-RFE, PCA (feature selection)	ANN, SVM	86% (ANN)
[19]	Depression vs. Control	PSD, Higuchi fractal dimension	Logistic Regression (LR)	92%
[20]	Depression vs. Control	Detrended Fluctuation Analysis (DFA), correlation dimension, higuchi fractal, and lyapunov exponent	LDA, K-nearest neighbor (KNN), LR	90% (LR)
[21]	MCI vs. Control	Variational Mode Decomposition (VMD)	KNN, non-dominated sorting genetic algorithm (NSGA)	99.81%
[22]	MCI vs. Control	Soft-decision score features	AdaBoost	91.97%
[10]	Schizophrenia vs. Control	Linear and nonlinear features, PCA (feature reduction)	SVM, LR, KNN, Random Forest (RF), Decision Tree (DT)	89% (SVM)
[23]	Schizophrenia vs. Control	Statistical + Wavelet features	LR, SVM, DT, RF, AdaBoost, and Gradient Boost	97.98% (DT)

**Table 2:** Demographic information of participants.

Group	Age (Mean ± Standard Deviation)	Female	Male	Total Volunteer
Control	40.3 ± 13.8	27	16	43
Depression	69.8 ± 14.9	28	-	28
Alzheimer	70.7 ± 8.5	21	19	40
Schizophrenia	41.4 ± 16.8	15	27	42
MCI	73.6 ± 5.5	14	14	28

**Table 3:** Electrode locations for EEG recordings.

Channel Number	1	2	3	4	5	6	7	8	9	10	11	12	13	14	15	16	17	18	19
	Fp1	Fp2	F3	F4	C3	C4	P3	P4	O1	O2	F7	F8	T3	T4	T5	T6	Fz	Cz	Pz

**Table 4:** Extracted features.

ID	Group	Feature Name	Abbreviation	Brief Explanation of Feature
1	Frequency axis measures	Frequency value	freqmax	The frequency value that gives the peak power value as a result of the power spectral density (PSD) analysis.
2		Power content	powmax	Maximum power value of PSD, indicating the dominant power in the signal
3		Spectral edge frequency	spedge	Spectral edge frequency indicates the frequency limit below which a certain percentage (in this case 50%) of the total spectral energy of a signal lies.
4	Entropy measures	Approximation Entropy	ApEn	A measure of the regularity and complexity of time-series data, with lower values indicating more regular patterns.
5		LogEnergy Entropy	logenergy	Quantifies the entropy or unpredictability based on logarithmic energy values of the signal
6		Permutation Entropy	PermEn	A measure of complexity that reflects the order and patterns in time-series data.
7		Tsallis Entropy	tsallis	A generalization of Shannon entropy that provides information about the complexity and structure in the data.
8		Renyi Entropy	renyi	A measure of entropy that generalizes the concept by focusing on different aspects of data distribution.
9		Spectral Entropy	spentropy	Entropy computed from the power spectral density, indicating the signal’s frequency-domain complexity.
10		Spectral entropy for dyadic levels	spentropDyd	Spectral entropy calculated over dyadic levels, offering insights into multi-scale signal complexity.
11	Hjorth parameters	Activity	activity	The variation in the time-series signal is related to its power and total energy.
12		Mobility	mobility	Measures the frequency variation in the signal, related to the ratio of the standard deviation of the first derivative.
13		Complexity	complexity	Quantifies the deviation from pure sine wave activity, assessing the signal’s frequency modulations.
14	Statistical measures	Kurtosis	kurt	Describes the “tailedness” of the probability distribution, indicating outlier presence in the signal.
15		Skewness	skew	Measures the asymmetry of the signal’s amplitude distribution, indicating if values are skewed towards higher or lower values.
16	Complexity measures	Lempel–Ziv complexity	lempel	A measure of complexity based on the compressibility of the signal; higher values indicate more irregularity.
17		Kolmogorov complexity	kolmogorov	A measure of the complexity of a signal, representing the length of the shortest possible description (algorithm) that can generate it.

**Table 5:** Selected feature numbers.

Classified Classes	Selected Feature Numbers	Total Feature Numbers Before Selection
Control–depression	86	19 channel × 34 features = 646 features
Control–AD	99	
Control–schizophrenia	100	
Control–MCI	90	
Depression–AD	79	
Depression–schizophrenia	91	
Depression–MCI	74	
AD–schizophrenia	108	
AD–MCI	88	
Schizophrenia–MCI	87	

**Table 6:** Algorithms’ running times for two-class classification (control–disease).

Training Time				
Disease	SVM	LDA	ANN	RF
Alzheimer	18.3566	5.3536	61.5140	0.5953
Depression	15.3165	5.4467	35.5072	0.3766
Schizophrenia	18.5279	5.4374	42.0707	0.6761
MCI	16.7928	5.3649	29.7070	0.1619
**Testing Time**				
Disease	SVM	LDA	ANN	RF
Alzheimer	0.0034	0.0021	0.0014	0.3098
Depression	0.0037	0.0021	0.0013	0.2496
Schizophrenia	0.0036	0.0026	0.0013	0.3622
MCI	0.0036	0.0020	0.0013	0.0896

**Table 7:** Binary classification results (control–disease).

Control–Alzheimer						
Method	Accuracy	Sensitivity	Specificity	Precision	Fscore	AUC
SVM	0.9250 ± 0.1054	0.9500 ± 0.1054	0.9000 ± 0.1748	0.9217 ± 0.1301	0.9296 ± 0.0976	0.9688 ± 0.0988
LDA	**1.0000 **± 0.0000	1.0000 ± 0.0000	1.0000 ± 0.0000	1.0000 ± 0.0000	1.0000 ± 0.0000	1.0000 ± 0.0000
ANN	0.8750 ± 0.1863	0.8750 ± 0.3173	0.8750 ± 0.2125	0.8963 ± 0.1637	0.9284 ± 0.1211	0.8750 ± 0.1863
RF	0.7208 ± 0.1498	0.7250 ± 0.2189	0.7200 ± 0.1636	0.7067 ± 0.1493	0.7060 ± 0.1818	0.7225 ± 0.1474
**Control–Depression**						
Method	Accuracy	Sensitivity	Specificity	Precision	Fscore	AUC
SVM	0.9589 ± 0.0945	0.9333 ± 0.1405	0.9750 ± 0.0791	0.9667 ± 0.1054	0.9467 ± 0.1167	0.9917 ± 0.0264
LDA	**0.9857 **± 0.0452	**1.0000 **± 0.0000	0.9800 ± 0.0632	0.9667 ± 0.1054	0.9800 ± 0.0632	**0.9900 **± 0.0316
ANN	0.9446 ± 0.0717	0.9667 ± 0.1054	0.9300 ± 0.1135	0.9167 ± 0.1361	0.9314 ± 0.0906	0.9483 ± 0.0686
RF	0.7339 ± 0.1186	0.5333 ± 0.2194	0.8550 ± 0.2088	0.8100 ± 0.2470	0.6055 ± 0.1534	0.6942 ± 0.1061
**Control–Schizophrenia**						
Method	Accuracy	Sensitivity	Specificity	Precision	Fscore	AUC
SVM	0.9403 ± 0.1010	0.9500 ± 0.1054	0.9300 ± 0.1135	0.9300 ± 0.1135	0.9389 ± 0.1054	0.9738 ± 0.0641
LDA	**0.9778 **± 0.0468	0.9600 ± 0.0843	1.0000 ± 0.0000	1.0000 ± 0.0000	0.9778 ± 0.0468	0.9800 ± 0.0422
ANN	0.9639 ± 0.0583	0.9750 ± 0.0791	0.9500 ± 0.1054	0.9600 ± 0.0843	0.9635 ± 0.0594	0.9625 ± 0.0604
RF	0.7278 ± 0.1386	0.6550 ± 0.2140	0.7800 ± 0.2486	0.7842 ± 0.2077	0.6962 ± 0.1774	0.7175 ± 0.1419
**Control–Mild cognitive impairement**						
Method	Accuracy	Sensitivity	Specificity	Precision	Fscore	AUC
SVM	0.9571 ± 0.0690	0.9333 ± 0.1405	0.9750 ± 0.0791	0.9750 ± 0.0791	0.9457 ± 0.0888	**1.0000 **± 0.0000
LDA	**0.9714 **± 0.0602	0.9667 ± 0.1054	0.9750 ± 0.0791	0.9750 ± 0.0791	0.9657 ± 0.0735	0.9708 ± 0.0623
ANN	0.9571 ± 0.0964	0.9333 ± 0.1405	0.9750 ± 0.0791	0.9667 ± 0.1054	0.9467 ± 0.1167	0.9542 ± 0.1010
RF	0.7304 ± 0.1589	0.4833 ± 0.2772	0.9000 ± 0.1748	0.8333 ± 0.2500	0.6265 ± 0.2058	0.6917 ± 0.1610

**Table 8:** Binary classification results (disease–disease).

Alzheimer–MCI						
Method	Accuracy	Sensitivity	Specificity	Precision	Fscore	AUC
SVM	0.9262 ± 0.0781	0.9500 ± 0.1054	0.9000 ± 0.1610	0.9400 ± 0.0966	0.9381 ± 0.0663	0.9833 ± 0.0351
LDA	**0.9690 **± 0.0655	0.9500 ± 0.1054	**1.0000 ± 0.0000**	**1.0000 ± 0.0000**	0.9714 ± 0.0602	0.9750 ± 0.0527
ANN	0.9262 ± 0.0781	0.9500 ± 0.1054	0.9000 ± 0.1610	0.9400 ± 0.0966	0.9381 ± 0.0663	0.9250 ± 0.0805
RF	0.7524 ± 0.1677	0.9000 ± 0.1291	0.5500 ± 0.3148	0.7538 ± 0.1541	0.8138 ± 0.1246	0.7250 ± 0.1845
**Alzheimer–Schizophrenia**						
Method	Accuracy	Sensitivity	Specificity	Precision	Fscore	AUC
SVM	0.8667 ± 0.1220	0.8500 ± 0.1748	0.8850 ± 0.1700	0.8950 ± 0.1462	0.8575 ± 0.1279	0.9238 ± 0.1256
LDA	0.9278 ± 0.0624	0.9250 ± 0.1208	0.9300 ± 0.1135	0.9400 ± 0.0966	0.9238 ± 0.0668	0.9275 ± 0.0629
ANN	0.9028 ± 0.1143	0.8500 ± 0.2415	0.9550 ± 0.0956	0.9600 ± 0.0843	0.8749 ± 0.1793	0.9025 ± 0.1145
RF	0.7181 ± 0.1058	0.7750 ± 0.1419	0.6550 ± 0.2443	0.7271 ± 0.1647	0.7307 ± 0.0832	0.7150 ± 0.1042
**Depression–Alzheimer**						
Method	Accuracy	Sensitivity	Specificity	Precision	Fscore	AUC
SVM	0.8810 ± 0.0635	0.9000 ± 0.1610	0.8750 ± 0.1318	0.8583 ± 0.1524	0.8571 ± 0.0797	0.9208 ± 0.1010
LDA	0.9429 ± 0.0738	0.9667 ± 0.1054	0.9250 ± 0.1208	0.9250 ± 0.1208	0.9371 ± 0.0828	0.9458 ± 0.0710
ANN	0.8238 ± 0.1118	0.7667 ± 0.3162	0.8750 ± 0.1318	0.8333 ± 0.1614	0.8201 ± 0.0871	0.8208 ± 0.1333
RF	0.7333 ± 0.1830	0.5500 ± 0.3518	0.8500 ± 0.1748	0.6917 ± 0.4045	0.7327 ± 0.1625	0.7000 ± 0.2068
**Depression–MCI**						
Method	Accuracy	Sensitivity	Specificity	Precision	Fscore	AUC
SVM	**0.9667 **± 0.0703	0.9667 ± 0.1054	0.9667 ± 0.1054	0.9750 ± 0.0791	0.9657 ± 0.0735	0.9889 ± 0.0351
LDA	0.9633 ± 0.0777	0.9333 ± 0.1405	**1.0000 ± 0.0000**	**1.0000 ± 0.0000**	0.9600 ± 0.0843	0.9667 ± 0.0703
ANN	0.9467 ± 0.0864	0.9333 ± 0.1405	0.9667 ± 0.1054	0.9750 ± 0.0791	0.9457 ± 0.0888	0.9500 ± 0.0805
RF	0.6700 ± 0.2457	0.7000 ± 0.3583	0.6667 ± 0.3143	0.6778 ± 0.2438	0.7061 ± 0.2347	0.6833 ± 0.2250
**Depression–Schizophrenia**						
Method	Accuracy	Sensitivity	Specificity	Precision	Fscore	AUC
SVM	0.9143 ± 0.0999	0.8833 ± 0.1933	0.9300 ± 0.1135	0.9083 ± 0.1493	0.8790 ± 0.1400	0.9467 ± 0.1068
LDA	0.9571 ± 0.0690	0.9500 ± 0.1581	0.9500 ± 0.1054	0.9500 ± 0.1054	0.9381 ± 0.1124	0.9500 ± 0.0874
ANN	0.9143 ± 0.0999	0.9167 ± 0.1800	0.9050 ± 0.1235	0.8833 ± 0.1532	0.8848 ± 0.1375	0.9108 ± 0.1102
RF	0.6857 ± 0.1475	0.4333 ± 0.3063	0.8600 ± 0.1696	0.6979 ± 0.2744	0.5863 ± 0.2020	0.6467 ± 0.1596
**Schizophrenia–MCI**						
Method	Accuracy	Sensitivity	Specificity	Precision	Fscore	AUC
SVM	0.8714 ± 0.1251	0.8167 ± 0.2540	0.9000 ± 0.1748	0.9017 ± 0.1622	0.8240 ± 0.1781	0.9833 ± 0.0351
LDA	0.9571 ± 0.0690	0.9333 ± 0.1405	0.9750 ± 0.0791	0.9750 ± 0.0791	0.9457 ± 0.0888	0.9542 ± 0.0747
ANN	0.7857 ± 0.2156	0.8000 ± 0.2811	0.7850 ± 0.2657	0.7767 ± 0.2682	0.7526 ± 0.2341	0.7925 ± 0.2143
RF	0.7429 ± 0.1622	0.6500 ± 0.2772	0.8100 ± 0.2171	0.7667 ± 0.2509	0.6543 ± 0.2015	0.7300 ± 0.1626

**Table 9:** Three-class classification results for the LDA algorithm (control–disease–disease).

Classess	Accuracy	Sensitivity	Specificity	Precision	Fscore	AUC
Cont-Depr-Alz	0.7583 ± 0.1495	0.7350 ± 0.1573	0.8808 ± 0.0735	0.7488 ± 0.1695	0.7576 ± 0.1540	**0.7840 **± 0.1939
Cont-Depr-MCI	0.7114 ± 0.1513	0.7294 ± 0.1449	0.8565 ± 0.0780	0.7250 ± 0.1513	0.7127 ± 0.1442	0.5702 ± 0.1262
Cont-Depr-Sch	0.7432 ± 0.1422	0.7333 ± 0.1468	0.8779 ± 0.0671	0.7672 ± 0.1280	0.7280 ± 0.1449	0.5325 ± 0.1116
Cont-Sch-Alz	0.7603 ± 0.1432	0.7583 ± 0.1445	0.8787 ± 0.0723	0.7938 ± 0.1494	0.7490 ± 0.1452	0.4484 ± 0.0788
Cont-Sch-MCI	0.7424 ± 0.1015	0.7489 ± 0.1080	0.8677 ± 0.0533	0.7853 ± 0.0939	0.7379 ± 0.1203	0.5421 ± 0.0977
Cont-MCI-Alz	**0.7932 **± 0.1041	0.7950 ± 0.0943	0.8950 ± 0.0575	0.8128 ± 0.1173	0.7863 ± 0.1015	0.5975 ± 0.1133

Cont = ‘Control’, Depr = ‘Depression’, Alz = ‘Alzheimer’, MCI = ‘Mild cognitive impairment’, Sch = ‘Schizophrenia’.

**Table 10:** Three-class classification results for the LDA algorithm (three disorders).

Classess	Accuracy	Sensitivity	Specificity	Precision	Fscore	AUC
Depr-Alz-MCI	0.8100 ± 0.0732	0.8028 ± 0.1051	0.8984 ± 0.0456	0.8698 ± 0.0537	0.8203 ± 0.0635	0.8925 ± 0.1131
Depr-MCI-Sch	**0.8467 **± 0.1088	0.8278 ± 0.1184	0.9208 ± 0.0537	0.8650 ± 0.1278	0.8296 ± 0.1216	**0.9128 **± 0.1090
Depr-Sch-Alz	0.6455 ± 0.1088	0.6428 ± 0.0994	0.8214 ± 0.0542	0.6678 ± 0.1161	0.6268 ± 0.1047	0.7298 ± 0.1536
Alz-MCI-Sch	0.7636 ± 0.1369	0.7450 ± 0.1636	0.8843 ± 0.0693	0.7600 ± 0.1519	0.7771 ± 0.1186	0.7875 ± 0.1602

Cont = ‘dontrol’, Depr = ‘depression’, Alz = ‘Alzheimer’, MCI = ‘mild cognitive impairment’, Sch = ‘schizophrenia’.

**Table 11:** Four-class classification results for the LDA algorithm.

Classess	Accuracy	Sensitivity	Specificity	Precision	Fscore
Cont-Depr-Sch-Alz	0.5146 ± 0.1148	0.5029 ± 0.1098	0.8388 ± 0.0391	0.5338 ± 0.1175	0.5148 ± 0.1044
Cont-Depr-Sch-MCI	0.5605 ± 0.1410	0.5575 ± 0.1503	0.8533 ± 0.0475	0.5971 ± 0.1802	**0.6122** ± 0.0647
Cont-Depr-Sch-CD	**0.5789 **± 0.1309	0.5389 ± 0.1427	0.8589 ± 0.0435	0.5475 ± 0.1559	0.5966 ± 0.1141
Cont-Sch-Alz-MCI	0.5617 ± 0.0888	0.5533 ± 0.0902	0.8534 ± 0.0305	0.6150 ± 0.0742	0.5701 ± 0.0856
Cont-Depr-Alz-MCI	0.5555 ± 0.1471	0.5296 ± 0.1436	0.8540 ± 0.0496	0.5429 ± 0.1670	0.6064 ± 0.1548
Depr-Sch-Alz-MCI	0.4857 ± 0.1171	0.4667 ± 0.1235	0.8272 ± 0.0383	0.4904 ± 0.1485	0.5410 ± 0.1033

Cont = ‘control’, Depr = ‘depression’, Alz = ‘Alzheimer’, MCI = ‘mild cognitive impairement’, Sch = ‘schizophrenia’, CD = ‘cognitive decline’. CD designates Alzheimer and MCI as the same class.

**Table 12:** Summary of related studies.

Study	Disorders Compared	Features/Methods	Classifier	Classes	Accuracy/Metrics
Huang et al. [70]	Depression vs. HC	SVM-RFE, Lasso, PCA	KNN	2	88.2% Acc
Shor et al. [30]	Schizophrenia, Depression, HC	p-adic spatio-temporal EEG analysis	–	2	AUC 99.89% (HC–Depr), 86.08% (Depr–Sch)
Jang et al. [71]	Schizophrenia, Depression, HC	N1, P3 amplitudes (P300 task)	LDA, SVM	2	Acc 71.31% (Sch–HC), 74.55% (Depr–HC), 59.71% (Sch–Depr)
Emre et al. [32]	Multiple disorders & HC	Frequency content	C5.0, RF, SVM, ANN	Multi	0.762–0.841 Acc
Sarisik et al. [31]	Schizophrenia, Depression, HC	Central alpha power, EphysAGE	SVM	2	Acc 72.7% (Sch–HC), 67% (Depr–HC), 63.2% (Sch–Depr)
Wang et al. [33]	Schizophrenia, Depression, HC	MUCHf-Net (CNN)	CNN	3	79.27% Acc
Cheng et al. [72]	NPMD, PMD, Schizophrenia, HC	Dynamic Functional Connectivity	RF	4	73.1% Acc
Hassanzadeh et al. [73]	AD, Schizophrenia, HC	ICA, Pearson correlation	KNN, RF, LR, SVM	2/3	Acc 89% (AD-Sch), 68% (AD-Sch-HC)
Akrofi et al. [74]	AD, MCI, HC	EEG features	KNN, MDA	3	84% Acc
McBride et al. [75]	AD, MCI, HC	Entropy, spectral power, complexity	SVM	3	85.4% Acc
Fiscon et al. [76]	AD, MCI, HC	Frequency features	DT	2/3	73.4% (MCI-AD-HC)–91.7% (HC-MCI) Acc
Pirrone et al. [26]	AD, MCI, HC	Power band differences	KNN, SVM, DT	2/3	75% (HC-MCI-AD)–97% (HC-MCI) Acc
Ieracitano et al. [77]	AD, MCI, HC	CWT + bispectrum	MLP	3	89.22% Acc
Oltu et al. [25]	AD, MCI, HC	DWT, PSD, coherence	Bagged Tree	3	96.5% Acc

HC = ’healthy control’, Depr = ‘depression’, AD = ‘Alzheimer’s disease’, MCI = ‘mild cognitive impairment’, Sch = ‘schizophrenia’, NPMD = ‘nonpsychotic major depression’, PMD = ‘psychotic major depression’.

## Data Availability

The data that support the findings of this study are openly available in [Dryad] at [https://doi.org/10.5061/dryad.8gtht76pw], reference number [34].

## Supplementary Materials

The following supporting information can be downloaded at: https://www.mdpi.com/article/10.3390/diagnostics15172132/s1, Supplementary File S1: groups with significant differences as a result of post hoc analysis; Supplementary File S2: Significant features and different groups.
